# Antibodies to the envelope protein protect macaques from SIVmac251 acquisition in an immunization regimen that mimics the RV-144 Thai trial

**DOI:** 10.1186/1742-4690-9-S2-O2

**Published:** 2012-09-13

**Authors:** G Franchini, P Pegu, S Gordon, B Keele, M Doster, Y Guan, G Ferrari, R Pal, MG Ferrari, S Whitney, L Hudacik, E Billings, M Rao, D Montefiori, D Venzon, C Fenizia, J Lifson, D Stablein, J Tartaglia, N Michael, J Kim

**Affiliations:** 1 NCI/NIH, Bethesda, MD, USA; 2 NCI-Frederick, Frederick, MD, USA; 3 Institute of Human Virology Maryland University School of Medicine, Baltimore, MD, USA; 4 Duke University, Durham, NC, USA; 5 Advanced Bioscience Laboratories, Rockville, MD, USA; 6 ABL, Rockville, MD, USA; 7 Advanced Biosciences Laboratories, Rockville, MD, USA; 8 Army Institute of Research, Silver Spring, MD, USA; 9 Duke University Medical Center, Durham, NC, USA; 10 Sanofi Pasteur Inc, Swifter, PA, USA; 11 Walter Reed Army Intitute of Research, Silver Spring, MD, USA; 12 Walter Reed Army Institute of Research, Sliver Spring, MD, USA

## Background

The canarypox vector ALVAC-HIV, together with the HIV gp120 envelope, has protected 31.2% of Thai heterosexual individuals from HIV acquisition in the RV144 HIV vaccine trial. This outcome was unexpected, given the limited ability of the ALVAC-HIV vaccine component to induce CD8+T-cell responses, and of the HIVgp120 envelope to elicit broad neutralizing antibodies.

## Methods

We vaccinated macaques with an immunization regimen that mimics the RV144 trial and exposed them to a mucosal dose of SIV_mac251_ that transmits few virus variants, similar to HIV transmission to humans.

## Results

Vaccination induced anti-envelope antibodies, modest CD4+ and CD8+ T-cell responses. One third of the vaccinated macaques were protected from SIV_mac251_ acquisition, whereas the remaining infected vaccinees progressed to disease. Vaccine induced SIV _mac251_ specific T-and B-cell responses were not different in protected or infected animals. The sera of the animals protected had higher avidity antibodies to the gp120 envelope protein, recognized the variable envelope region V2, and reduced SIV_mac251_ infectivity in cells that express high level of α4β7, suggesting a functional role to antibodies to V2.

## Conclusion

The SIV _mac251_ infection macaque faithfully reproduces results in humans, and is instrumental in the development of more efficacious vaccines for HIV.

